# Trophic Dynamics of Deep-Sea Megabenthos Are Mediated by Surface Productivity

**DOI:** 10.1371/journal.pone.0063796

**Published:** 2013-05-17

**Authors:** Samuele Tecchio, Dick van Oevelen, Karline Soetaert, Joan Navarro, Eva Ramírez-Llodra

**Affiliations:** 1 Institut de Ciències del Mar, Consejo Superior de Investigaciones Científicas, Barcelona, Spain; 2 Royal Netherlands Institute for Sea Research, Yerseke, The Netherlands; Universidade Federal do Rio de Janeiro, Brazil

## Abstract

Most deep-sea benthic ecosystems are food limited and, in the majority of cases, are driven by the organic matter falling from the surface or advected downslope. Species may adapt to this scarceness by applying a wide variety of responses, such as feeding specialisation, niche width variation, and reduction in metabolic rates. The Mediterranean Sea hosts a gradient of food availability at the deep seafloor over its wide longitudinal transect. In the Mediterranean, broad regional studies on trophic habits are almost absent, and the response of deep-sea benthos to different trophic conditions is still speculative. Here, we show that both primary and secondary production processes taking place at surface layers are key drivers of deep-sea food web structuring. By employing an innovative statistical tool, we interpreted bulk-tissue δ^13^C and δ^15^N isotope ratios in benthic megafauna, and associated surface and mesopelagic components from the 3 basins of the Mediterranean Sea at 3 different depths (1200, 2000, and 3000 m). The trophic niche width and the amplitude of primary carbon sources were positively correlated with both primary and secondary surface production indicators. Moreover, mesopelagic organic matter utilization processes showed an intermediate position between surface and deep benthic components. These results shed light on the understanding of deep-sea ecosystems functioning and, at the same time, they demand further investigation.

## Introduction

Heterotrophic, bottom-up controlled, food webs are the most abundant ecosystem structure on the planet; i.e. they are the most frequent food webs found in the deep sea, which is the largest biome on Earth [1], [2]. Deep-sea systems thrive on a downward flux of particulate organic matter, the *marine snow*, which provides the main food source for the benthos [3]. Recent advances in benthic ecology identified that deep-sea food webs present a complex trophic structure, with a high number of trophic levels and various processes of niche adaptation [4]–[7]. Organic matter input and its availability at the seafloor have also been shown to control benthic standing stock, community composition, and diversity [8], [9]. This process of organic matter deposition – its magnitude decreasing with increasing depth – is essential in regulating how species interact with food sources and between them [10].

**Figure 1:**
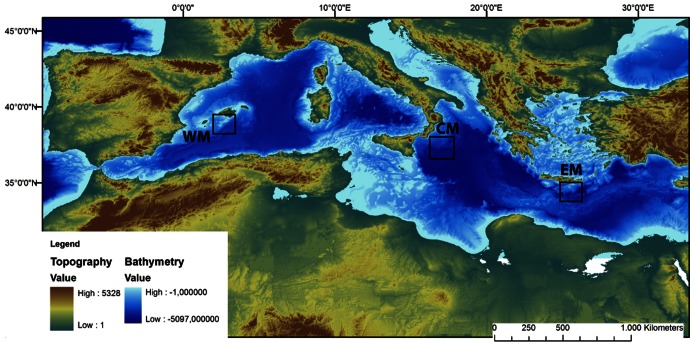
Bathymetric map of the study area. Map of the Mediterranean Sea, with bathymetry and showing the location of sampling sites. WM: Western Mediterranean (southern Balearic), CM: Central Mediterranean (western Ionian), EM: Eastern Mediterranean (south of Crete).

The Mediterranean Sea is geographically divided into three basins (Figure 1): the western, central and eastern, with important variations in primary productivity and organic matter availability between them [11]. The eastern basin is the most oligotrophic area in terms of organic matter input to bathyal depths [12] while, in the western basin, high fluvial inputs, increased surface productivity and other mesoscale oceanographic events generate a higher quantity of organic matter reaching the deep seafloor [13], [14]. It follows that the carbon fluxes of the western basin at similar depths are two orders of magnitude higher than those in the eastern basin [11]. The central basin presents intermediate environmental conditions between the west and the east, and its continental slope hosts a diverse benthic megafaunal community, more similar to the one present in the western basin [15]. This gradient spans the entire Mediterranean region and thus provides an interesting benchmark to test for macro-ecological patterns in numerous processes such as biodiversity and trophic relationships.

In marine systems, depth is also considered another major driver of benthic processes, such as species distribution [16]. Depth provides an additional gradient of food availability and quality at the bottom, because the amount of degradation of organic matter during its fall is correlated with the height of the water column [17]. Generally speaking, water depth thus negatively correlates with energy availability at the seafloor [10]. In all, the Mediterranean Sea offers two dimensions of organic matter input, i.e. bathymetric and longitudinal, that can be used to study the control of organic matter input on an important component of the benthic food web, namely megabenthos.

Megabenthic communities of the continental margins and deep basins of the Mediterranean Sea, dominated by fishes and decapod crustaceans in terms of abundance and biomass, present strong biomass reductions that follow the gradients of productivity and food arrival at the seafloor [18]. However, how megafauna respond to a gradient in organic matter input is still a matter of speculation. Studies conducted until now have not managed to disclose the actual process modulating these adaptations [19], [20].

Deep-sea Mediterranean studies on dietary habits, niche overlap, and more generally, trophic dynamics, were conducted traditionally by stomach content analyses [19], [21]. However, existing collection methods for fish fauna at high bathyal depths do not permit the retrieval of an acceptable number of useful dietary samples, mostly because of the high number of everted stomachs caused by decompression shock. Other issues impair completeness in dietary studies of deep-sea species, namely (i) extremely scant information about ingestion rates, (ii) overestimation of preys that leave hard structures such as fish otoliths or cephalopod beaks, and (iii) relatively low number of samples for diet descriptions to be credible.

In the last decades, the use of stable isotopes (especially δ^13^C and δ^15^N values) has become an important tool in marine ecosystem studies [22]. Because of the isotopic discrimination in metabolic processes, the nitrogen isotope ratio increases by 2.3 to 5‰ with each trophic step, while the carbon isotope ratio of a consumer is only slightly enriched (between 0 and 1.5‰) with respect of that of its food [23]–[25]. As a result, the carbon isotope is an indicator of the sources of organic matter, while the nitrogen isotopes ratio informs on the trophic position of the individual in the food web [26]–[28].

As carbon and nitrogen stable isotopes are indicators of both the sources of organic matter and the structure of the food web, they provide two dimensions of observation that are linkable with the trophic niche concept [29]. A trophic niche is defined as an n-dimensional hyper-volume representing the *role* of a particular species in the trophic web [30]. Carbon and nitrogen stable isotope ratios are usually plotted in an isotopic bi-space, in which the variability has been demonstrated to be an indicator of the trophic niche width of the analysed individuals [31]. Quantitative metrics based on the geometric distribution of carbon and nitrogen isotopes ratios in the 2-dimensional space, have been recently developed to evaluate community-wide trophic dynamics such as vertical structuring, species packaging and niche diversification [32], [33]. Species that adapt to organic matter scarcity by specialising would show a decrease in their occupied area in the bi-space, and an increase of their distance from the other species (i.e. reduced packaging). By contrast, generalisation should be evidenced by a broadening of the carbon isotope signature, and by an increase of their occupied area.

In the present study, carbon and nitrogen stable isotopic analyses were applied to address 2 objectives: (a) to reveal how trophic niches develop along depth and productivity gradients in the deep Mediterranean Sea, and (b) to reveal the contribution of marine snow in diets of deep-sea food webs. The results will strengthen the connections between surface processes and benthic trophic niche adaptations.

## Materials and Methods

### Sampling Procedures

All samples were collected in June 2009 in the three basins of the deep Mediterranean Sea (western, central, and eastern basins) at three depths (1200, 2000, and 3000 m) in each of the three basins (Figure 1). In the western basin, we sampled the southern Balearic region (code W), in the central basin we sampled the western Ionian Sea (code C), while the southern Cretan Sea was considered for the eastern basin (code E). Permission for sampling was obtained by the Spanish Ministry of Education and Science for the W site, and by the Spanish Ministry of External Affairs in collaboration with the consulates of Italy and Greece for the C and E sites.

Nektobenthic and benthopelagic megafauna were captured with an otter-trawl Maireta system (OTMS), while strictly benthic species were captured with an Agassiz dredge [1], [34]. Both gears employed a stretched net mesh size of 12 mm, and were used in parallel because of their sampling complementarity [18]. The duration of the trawls ranged between 0.7 and 2.0 h. Every individual collected was identified to species level. Muscle samples without skin (in the case of fishes) and without exoskeleton (in the case of crustaceans) from individuals of all collected species were immediately retrieved after sorting on board and were immediately frozen at -20°C until their isotopic determination. All individuals arrived on the deck already dead. They were treated according to the current Spanish national regulations on vertebrate research, which do not require any specific permit to work with trawl samples, and procedures were internationally approved by the European Science Foundation (ESF). None of the collected species is currently in protection status.

At the same sampling stations, pelagic microplankton (size range: 53–200 µm) and mesozooplankton (size range: 200–2000 µm) samples were also collected by using WP2 plankton nets in vertical hauls from 200 m depth to the surface. Plankton samples were filtered on board, on G/FC glass microfiber filters. Macroplankton from the deep scattering layer (identified by echo sounding) was additionally collected with an Isaaks-Kidd Midwater Trawl (IKMT), and selected species from each sample were pooled together. Similarly to megafauna samples, all plankton samples were stored frozen at −20°C until their isotopic determination.

### Isotopic Analysis

Stable isotopes analyses were conducted at the dedicated facility of the Netherlands Institute for Sea Research (NIOZ), in Yerseke, The Netherlands. In the laboratory, all samples collected were freeze-dried for 48 hours and grounded to a fine powder. Drop-by-drop acidification with diluted HCl (0.1 M) was performed only on suprabenthos and plankton samples, with no water rinsing afterwards, to remove the calcium shells [35]. Stable isotope ratios ^13^C/^12^C and ^15^N/^14^N, and organic carbon and nitrogen content were measured simultaneously on a Flash EA 1112 coupled to a DeltaV Advantage IRMS (Thermo Electron Instruments). Monitoring of CO_2_ (m/z = 44 and 45) and N_2_ (m/z = 28 and 29) ion currents of samples against standards with known C and N content allowed accurate measurement of organic carbon and nitrogen contents to determine C/N ratios. δ^13^C_org_ and δ^15^N_tot_ values are expressed relative to Vienna Pee-Dee Belemnite and air and normalized to δ^13^C and δ^15^N of USGS40 and USGS41. All measurements were corrected for blanks, and NIOZ laboratory standards were run alongside each 96-samples plate. Values of δ^13^C and δ^15^N were pooled by species, and considered separately in each of the sampled sites.

### Isotopic Metrics and Trophic Levels

Due to their differences in displacement capabilities and behaviour, the analysed megafauna was split between fish and invertebrate (mainly crustaceans) species. The community-wide metrics applied are described and validated in detail by Layman et al. [32]. Briefly, the following indices were considered:

- δ^15^N range (dNR): expresses the distance between the most ^15^N-enriched and the most ^15^N-depleted samples in the community, and is an indicator of its vertical structuring (i.e. trophic length).
- δ^13^C range (dCR): the equivalent of dNR considering ^13^C, provides an indicator of the diversity of basal food resources.
- Total convex hull area (TA): the area of the smallest convex polygon containing all species in the isotopic bi-space. TA is correlated with the total niche amplitude of the food web.
- Mean distance to centroid (CD): the average Euclidean distance of each species to the δ^13^C - δ^15^N centroid (which is the mean δ^13^C and δ^15^N value of the entire food web). It correlates positively with the trophic niche amplitude and with the spacing between species.

Community-wide indices were calculated using the *SIAR* package for the R statistical language (“Stable Isotope Analysis in R”; [36]). As sample sizes differed among sites, a Bayesian approach was adopted to propagate uncertainty in the mean values of the metrics using 10^4^ randomly calculated communities [33]. Bayesian isotopic ellipses (SEA) for each site were calculated, considering only benthic megafauna.

The trophic level (TL_consumer_) of each individual was estimated using the equation:
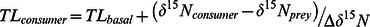
δ^15^N_prey_ and δ^15^N_consumer_ were, respectively, the isotopic values of microplankton and individual fish or crustacean obtained in the present study (at each site). A basal trophic level (TL_basal_) of 1.5 was applied, assuming that microplankton (mostly composed by phytoplankton) possesses a trophic level between 1 of the primary producers and 2 of micro- and mesozooplankton [37]. A value of Δδ^15^N = 3.5‰ was used as the isotopic discrimination factor for nitrogen [38].

### Environmental Variables

A series of environmental variables, collected at exactly the same sites than the megafauna samples, were recorded also. Values for these variables were taken directly from the study by Tecchio et al. [18], and the variables considered were: benthic temperature (°C), benthic salinity, benthic turbidity (Formazin turbidity units, FTU), surface fluorescence (relative fluorescence units, RFU), sediment grain size (% of coarse fraction, >63 µm), sediment particulate organic carbon (POC, % of mass), microplankton biomass (mg m^−3^), and mesozooplankton biomass (mg m^−3^). The mean values of fluorescence between 0 and 150 m depth were used as an estimator of surface primary production, as the data were collected with the same CTD and protocols throughout the sampling phase. Biomass of microplankton and mesozooplankton was integrated between 0 and 200 m depth in the water column.

### Statistical Analyses

Two-way ANOVA tests and pair-wise Tukey’s HSD post-hoc tests were applied to test for differences between depths and sites simultaneously in δ^13^C, δ^15^N and trophic level values. Non-parametric Kruskal-Wallis tests were used to compare means of isotopic metrics between depths and basins. Spearman rank correlation tests were used to identify correlations between environmental variables and δ^13^C and δ^15^N means, and isotopic metrics.

## Results

**Figure 2:**
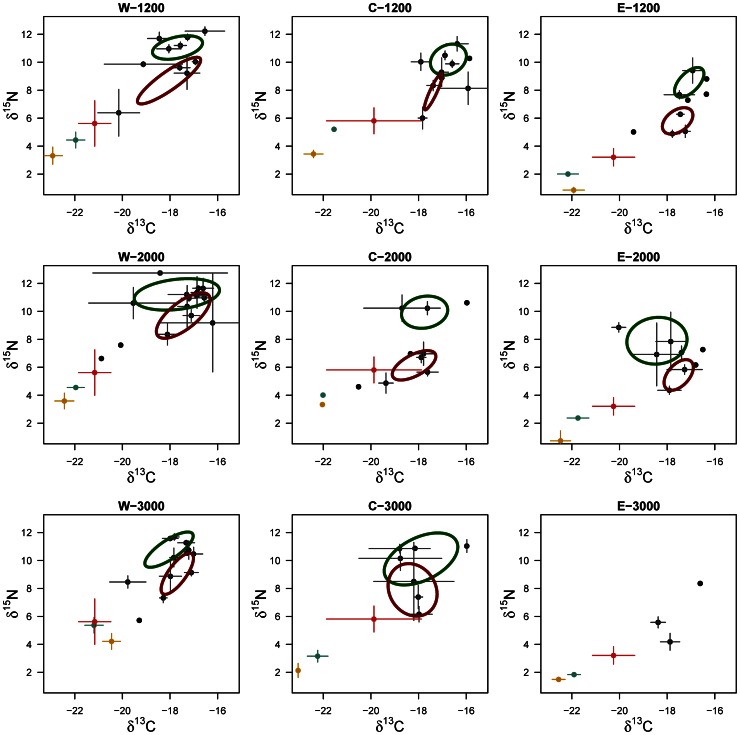
Isotopic biplots for the deep Mediterranean Sea. Mean δ^13^C and δ^15^N values for megafauna and plankton components in the 3 basins of the Mediterranean Sea at 3 different depths. Isotopic ellipses for each site are drawn separately for fishes (green) and crustaceans (brown) of benthic megafauna. Colour codes for points: yellow – microplankton, red – mesozooplankton, green – mesopelagic macroplankton, grey – demersal megafauna.

The collected megafauna was mainly composed by Actinopterygii fishes and decapod crustaceans, and the number of specimen analysed in each site ranged from 33 to 108 (Table S1). Microplankton and mesozooplankton occupied the lower portion of the bi-isotopic space in all sites, segregated from the species in the benthic domain (Table 1 and Figure 2). Mesozooplankton values of δ^15^N were higher than microplankton values in the same basin, showing the natural isotopic enrichment with trophic level (Figure 2). This pelagic enrichment was not visible at the Eastern site at 1200 m depth, which could be caused both by the reduced number of samples and by their location: samples were collected both in the north (1200 m depth) and south (2000 and 3000 m depth) of the island of Crete, thus with potentially two differing food input regimes. Macroplankton from the deep scattering layer was always positioned between the surface planktonic components and the deep benthos isotopic values (Figure 2).

**Table 1:** Isotopic parameters for pelagic and benthic compartments.

	WM-1200		WM-2000		WM-3000	CM-1200	CM-2000	CM-3000	EM-1200	EM-2000	EM-3000
Plankton											
** Microplankton δ^13^C**	−22.93±0.41		−22.44±0.40		−20.45±0.38	−22.41±0.39	−22.04	−23.06±0.03	−21.92±0.44	−22.48±0.42	−22.55±0.27
** Microplankton δ^15^N**	3.31±0.62		3.59±0.56		4.21±0.57	3.44±0.26	3.34	2.13±0.50	0.85±0.20	0.74±0.71	1.49±0.08
** Mesozooplankton δ^13^C**	−21.96±0.38		−21.96±0.36		−21.19±0.39	−21.54±0.09	−22.01±0.07	−22.24±0.43	−22.16±0.44	−21.74±0.46	−21.91±0.27
** Mesozooplankton δ^15^N**	4.44±0.57		4.56±0.14		5.37±0.56	5.21±0.15	4.01±0.04	3.16±0.42	2.01±0.08	2.37±0.01	1.84±0.04
** DSL Macroplankton δ^13^C**	−21.16±0.67					−19.88±1.99			−20.52±0.88		
** DSL Macroplankton δ^15^N**	5.63±1.63					5.81±0.93			3.21±0.62		
Community metrics											
** δ^15^N range (dNR)**	7.42	6.33		6.13		6.68	7.04	5.98	5.43	6.21	4.94
** δ^13^C range (dCR)**	6.13	7.85		3.83		3.28	4.55	4.94	3.15	3.86	2.03
** Total hull area (TA)**	24.79	35.87		12.84		12.87	22.95	20.83	8.47	15.71	3.90
** Mean distance to centroid (CD)**	1.52	1.78		1.60		1.45	2.02	2.28	1.55	1.83	1.19
** Mean nearest neighbour distance (NND)**	0.43	0.44		034		0.35	0.50	0.72	0.32	0.48	0.86

Isotopic ratios (mean ± S.D.) for carbon and nitrogen in microplankton, mesozooplankton, and macroplankton of the deep scattering layer (DSL, available only by basin) and values of calculated community metrics in the 3 zones of the Mediterranean Sea (Western, Central, and Eastern basins) at 3 different depths.

δ^13^C values did not differ significantly between the three basins, when considering the whole assemblage (2-way ANOVA, F_2,353_ = 0.48, p = 0.61). δ^13^C values showed significant differences between the shallower site (1200 m depth) and the deeper layers of 2000 to 3000 m depth (Tukey HSD, p = 0.006), which grouped together (Tukey HSD, p = 0.06). Considering assemblages as a whole, δ^15^N values significantly changed between the 3 basins, decreasing from the Eastern to the Western basins (2-way ANOVA, F_2,353_ = 52.46, p<0.001), while δ^15^N values did not differ significantly between depths (F_2,353_ = 52.46, p = 0.11) in any basin (depth × basin interaction; F_2,353_ = 1.78, p = 0.13). Fish δ^13^C values showed similar values between basins (F_2,185_ = 0.35, p = 0.71) and differed significantly between the shallower site (1200 depth) and the deeper layers of 2000 to 3000 m (F_2,185_ = 7.61, p = 0.001; Tukey HSD, p<0.05). Fish δ^15^N values showed similar values between depths (F_2,185_ = 52.46, p = 0.12) and were significantly higher in the Western basin, than in both the Central and Eastern basins (F_2,185_ = 0.12, p<0.0001; Tukey HSD, p<0.05 for W-C and p<0.001 for W-E). Crustacean δ^13^C values did not differ between basins (F_2,168_ = 1.11, p = 0.33) and depths (F_2,168_ = 2.31, p = 0.11). Crustacean δ^15^N mean values were significantly higher in the Western basin, than in both the Central and Eastern basins (F_2,168_ = 39.32, p<0.0001; Tukey HSD, p = 0.005 for W-C and p<0.001 for W-E), and did not differ between depths (F_2,168_ = 0.48, p = 0.66).

The estimated TL of the whole assemblage did not differ significantly between depths (F_2,119_ = 0.52, p = 0.72). However, the TL of the western basin were significantly higher than the TL of Central and eastern basins (F_2,119_ = 4.58, p = 0.01; Tukey HSD, p = 0.01). The estimated trophic level (TL) of fish assemblages did not show any significant differences with depth (F_2,64_ = 0.49, p = 0.61) and regions (F_2,64_ = 2.08, p = 0.13). The TL of the crustacean assemblages did not differ between depths (F_2,55_ = 0.17, p = 0.95) but was higher at the western and eastern basins than those of the central basin (F_2,55_ = 0.71, p = 0.95; Tukey HSD, p<0.05).

Community-wide metrics (Table 1) were calculated with both traditional (exact, no variability) and Bayesian methods. Values of all metrics obtained by Bayesian estimation showed no significant differences between sites (Kruskal-Wallis test, p ∼ 1.00 for all contrasts). δ^15^N ranges (dNR) showed the highest values in the Western Mediterranean shallowest site (W-1200, dNR = 7.42‰), while in the other sites they ranged from 5.43‰to 7.04‰.

Ranges of δ^13^C (dCR) for benthic megafauna were comparable between sites, with all Bayesian ellipses contained between values of −19‰ and −16.5‰. Values of dCR ranged from 3.15‰ to 7.85‰ across the whole Mediterranean Sea. By contrast, the absolute values of δ^15^N were lower in the Eastern basin (3.42‰ –10.26‰) than in the other basins (Western basin was 7.00‰ –12.95‰, Central was 4.03‰ –19.87‰). Values of the mean centroid distance (CD) were always higher in the deepest sites than in the slope sites, suggesting an increase of the distance of species niches with increasing depth. This can be related to the difference in the TA, which, in the central basin, was the highest of the dataset.

The area of Bayesian isotopic ellipses did not show any appreciable pattern with depth and basin, although when considering fish alone, areas were the lowest in the shallowest sites of each basin (i.e. at 1200 m depth, see Figure S1). The same is true for the traditional equivalent, the total convex hull area (TA). As these values did not include a variance expression, statistical testing was not possible; however, we can consider TA and the area of Bayesian ellipses to convey the same ecological meaning.

**Figure 3:**
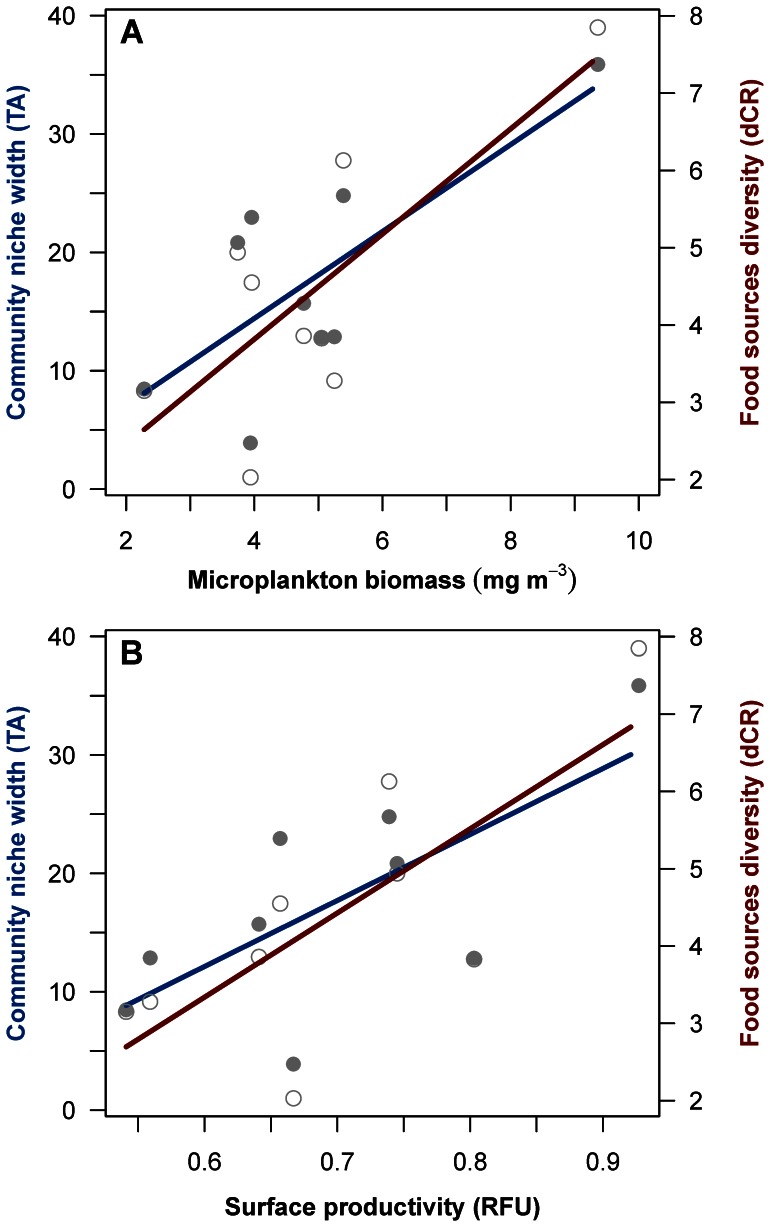
Surface modulation of deep trophic dynamics. Scatterplots showing the correlation between surface microplankton biomass (A) and surface productivity (B, estimated by fluorescence) against benthic trophic parameters: community niche width (TA, filled circles) and food sources diversity (dCR, open circles).

Pearson correlation tests yielded no significant relationships between SEA ellipse areas of each depth and locality and any environmental variable. The total hull area of the assemblages (TA) and the range of dCR of each depth and site were positively correlated with both surface microplankton biomass (TA: R^2^ = 0.55, p = 0.02. dCR: R^2^ = 0.58 and p = 0.01; Figure 3) and surface fluorescence (TA: R^2^ = 0.49, p = 0.03; dCR: R^2^ = 0.57, p = 0.01; Figure 3).

## Discussion

The Mediterranean Sea presents a distinct environment in each of its three basins, which renders it particularly interesting to test ecological hypotheses across environmental gradients [11], [12]. The continental slope areas in the Mediterranean are considered oceanographically dynamic, influenced by local-scale surface events such as river input and coastal atmospheric events [14], [39]. By contrast, the deepest areas considered in the present study (2000–3000 m depth) are subject to a considerably lower nutrient input and are thus more oligotrophic [13], [40]. This factor, coupled with the longitudinal gradient, leads to two axes of food availability, which have been addressed here.

The collected megafauna was a fair representation of the majority of the species in the deep Mediterranean Sea, both in terms of species and densities. The number of specimens analysed in each site was proportional to the decreasing gradient of megafaunal density found in the Mediterranean along both depth and west-to-east axes [15], [18]. The species analysed included nektobenthic fishes (such as Macrourids and Morids), species that perform vertical benthic-pelagic migrations (e.g. the shrimps *Acanthephyra spp.* and *Aristeus antennatus*, and various components of macroplankton such as myctophids and gonostomatiids) and less-mobile and sessile species, which are strictly connected to the sea floor (i.e. the reptantian crustaceans and non-crustacean invertebrates). Thus, the deep community analysed here can be considered an image of the benthic domain and of the overlying nektobenthic compartment.

The species that perform wide-ranging vertical migrations in the water column (i.e. the benthopelagic fishes and decapod crustaceans) play a major role in the *downward benthopelagic coupling*: the transfer processes from the pelagic domain to the benthos, and the responses of the latter [41]. As stated above, megafauna assemblages of the deep Mediterranean Sea primarily consist of nektobenthic and benthopelagic species, mainly fishes and decapod crustaceans [42]. Strictly benthic species (such as many molluscs and other low-motility non-crustacean invertebrates) are speciose but not abundant in the deep Mediterranean [15], [43]. The predominance of benthopelagic species may enhance levels of carbon transfer along the water column and towards the deep seafloor, with respect to the outer Atlantic and, thus, strengthen the downward coupling. In addition, the presence of nektobenthic species also increases the quantity of carbon laterally transported from the adjacent deep seafloor [44].

Deep benthic systems in the Mediterranean Sea are mainly linked to surface productivity, both primary and secondary, and to mesopelagic processes [18], [45]. In deep-basin areas, the main input of nutrients is performed by direct vertical sinking of organic carbon, while, on continental slopes, lateral advection and riverine input processes can also contribute significantly to the quantity of carbon reaching the seafloor [46]. In all sites examined in the present study, the isotopic positions of planktonic components (i.e. microplankton, mesozooplankton, and deep macroplankton) were evidently connected. This indicates a strong link, both in carbon sourcing and feeding relations, between the water-column domain and the demersal compartment. Furthermore, the positioning of the deep-layer macroplankton between surface and benthic components indicates a direct participation of this mesopelagic assemblage in the vertical organic matter sinking.

The microplankton fraction, as sampled in this study, is constituted by both phytoplankton and zooplankton species [37]. The amplitude of benthic community niche and the spectrum of carbon sources were positively correlated with both microplankton biomass and surface fluorescence, the latter being an indicator of primary production. Phytodetritus arrival at the deep sea plays a major role in well-studied areas of the northeast Atlantic Ocean [47], [48] or the Pacific Ocean [49], [50], where deposit feeders (such as holothurians which primarily feed on phytodetritus) dominate the benthic community. In the deep Mediterranean Sea, megafauna has been observed ignoring patches of phytodetritus that were experimentally delivered to the seafloor [51]. It is thus not clear how the highly-mobile benthic megafauna in the Mediterranean are adapted to process the phytodetritus input from the surface. In our case, the diversity of basal resources (estimated by the δ^13^C range, dCR) varied between depths and basins relative to the levels of primary and secondary surface productions. This may reflect slightly different sources of organic matter from the surface, generating a wider spectrum of types of carbon reaching the deep seafloor and, consequently, a wider niche space to be filled.

Two distinct carbon pathways were observed especially in the Western basin (see Figure 2). The more carbon-light pathway consists mainly of benthopelagic species (i.e. the smooth-head *Alepocephalus rostratus*), exploiting suprabenthos and gelatinous plankton, especially the jellyfish *Pelagia noctiluca* [21]. The other pathway comprises demersal species and includes all macrourid fish and benthic crustaceans. This suggests a split of the deep benthic food-web at the suprabenthic level, with a benthic detritus-based chain and a more pelagic-linked one. This phenomenon has been observed also in the deep Pacific Ocean [52]. It seems that *A. rostratus* is not constrained to feeding in the benthic food web but rather it is short-circuiting the bentho-pelagic coupling, feeding directly on plankton migrating from the water column. High quantities of gelatinous plankton that reach the lower slope [53] may be the reason for the dominance of *A. rostratus* in the demersal community between 1200 and 1350 m depth, in the western Mediterranean basin.

Also noteworthy are the high levels of carbon isotope enrichment with shifts to more positive values in the benthic megafauna with respect to basal components (i.e. surface zooplankton and mesopelagic macroplankton). The enrichment per trophic level stands on the higher end of the ranges usually considered in the literature [54]. An explanation of this phenomenon might be that, as particulate organic matter (POM) sinks in the water column, pelagic components and bacteria perform a metabolic degradation that shifts the carbon isotopic ratio to heavier levels [55]. If this is the case, then it would be accentuated by the high and constant water temperatures found in the Mediterranean Sea below 200 m depth (13–14°C), which increase the levels of prokaryotic degradation of organic matter [56]. This enrichment was not found in other studies in the deep Mediterranean [51], [57]. In our dataset, C:N values for fishes were generally low, thus also suggesting an increased utilization of isotopically-light compounds such as fatty acids, e.g. for reproduction.

The isotopic bi-space did not show any particular pattern over depth and longitude, neither with fishes nor with crustaceans. These results confirm the complexity of the food webs of the deep benthonic and supra-benthonic communities, and that these are not only strictly dependent by vertical detritus input. It is still a matter of speculation whether the changing trophic conditions over large geographic scales may modulate the response of the whole deep-sea benthic communities. In a study conducted at the Porcupine Abyssal Plain in the deep Atlantic ocean, it is explained that competition may be reduced by either increasing niche specialization or by vertically expanding the trophic structuring [4]. In the case of the Mediterranean Sea, it remains to be proven whether the increase in generalist trophic habits of the benthos would help to reduce competition for resources.

### Conclusions

Deep-sea ecosystem structure and functioning have been related to seasonality, mainly determined by intra-annual variations of surface primary productivity and climate-driven atmospheric events. This is the first study to directly address the patterns of niche width in the deep-sea benthos over such a large spatial scale. We conclude that (i) no clear pattern of trophic niches can be observed over large spatial scales for the deep Mediterranean megabenthos, and (ii) both surface primary and secondary productions clearly modulate the trophic niche width and the amplitude of primary carbon sources in the deep Mediterranean megabenthos.

## Supporting Information

Figure S1 **Benthic community niche widths in the deep Mediterranean Sea.** Area of Bayesian isotopic ellipses for demersal fishes (A) and crustaceans (B) in the 3 basins of the Mediterranean Sea (WM, CM, EM) at 3 different depths (1200, 2000, and 3000 m). Black dots represent the simulated median. Boxes represent the 50%, 75% and 95% confidence intervals (dark grey to light grey, respectively).
(TIF)

Table S1 **Carbon and nitrogen isotope ratios of benthic megafauna.** Values of δ^13^C and δ^15^N for all sampled benthic megafauna in the deep Mediterranean Sea (Mean ± S.D.), along with the measured carbon/nitrogen ratio (C:N) and the number of analysed specimen.
(DOC)

## References

[1] Gage JD, Tyler PA (1991) Deep-sea biology: a natural history of organisms at the deep-sea floor. Cambridge: Cambridge University Press. 504 p.

[2] Ramírez-Llodra E, Brandt A, Danovaro R, De Mol B, Escobar E, et al (2010) Deep, diverse and definitely different: unique attributes of the world’s largest ecosystem. Biogeosciences 7: 2851–2899.

[3] Polunin NVC, Morales-Nin B, Pawsey WE, Cartes JE, Pinnegar JK, et al (2001) Feeding relationships in Mediterranean bathyal assemblages elucidated by stable nitrogen and carbon isotope data. Mar Ecol Prog Ser 220: 13–23.

[4] Iken K, Brey T, Wand U, Voigt J, Junghans P (2001) Food web structure of the benthic community at the Porcupine Abyssal Plain (NE Atlantic): a stable isotope analysis. Prog Oceanogr 50: 383–405.

[5] Jeffreys RM, Wolff GA, Murty SJ (2009) The trophic ecology of key megafaunal species at the Pakistan Margin: evidence from stable isotopes and lipid biomarkers. Deep-Sea Res I 56: 1816–1833.

[6] Madurell T, Fanelli E, Cartes JE (2008) Isotopic composition of carbon and nitrogen of suprabenthic fauna in the NW Balearic Islands (western Mediterranean). J Mar Syst 71: 336–345.

[7] Tecchio S, Coll M, Christensen V (2013) Company JB, Ramírez-Llodra E, et al (2013) Food web structure and vulnerability of a deep-sea ecosystem in the NW Mediterranean Sea. Deep-Sea Res I 75: 1–15.

[8] Danovaro R, Gambi C, Lampadariou N, Tselepides A (2008) Deep-sea nematode biodiversity in the Mediterranean basin: testing for longitudinal, bathymetric and energetic gradients. Ecography 31: 231–244.

[9] Smith KL, Ruhl HA, Bett BJ, Billett DSM, Lampitt RS, et al (2009) Climate, carbon cycling, and deep-ocean ecosystems. Proc Natl Acad Sci USA 106: 19211–19218. 19901326 10.1073/pnas.0908322106 PMC2780780

[10] Levin LA, Etter RJ, Rex MA, Gooday AJ, Smith CR, et al (2001) Environmental influences on regional deep-sea species diversity. Annu Rev Ecol Evol Syst 32: 51–93.

[11] Danovaro R, Dinet A, Duineveld G, Tselepides A (1999) Benthic response to particulate fluxes in different trophic environments: a comparison between the Gulf of Lions–Catalan Sea (western-Mediterranean) and the Cretan Sea (eastern-Mediterranean). Prog Oceanogr 44: 287–312.

[12] Azov Y (1991) Eastern Mediterranean - a marine desert? Mar Pollut Bull 23: 225–232.

[13] Margalef R, editor (1985) Western Mediterranean. Oxford: Pergamon Press. 363 p.

[14] Company JB, Puig P, Sardà F, Palanques A, Latasa M, et al (2008) Climate influence on deep sea populations. PLOS ONE 3: e1431. 18197243 10.1371/journal.pone.0001431 PMC2174526

[15] Tecchio S, Ramírez-Llodra E, Sardà F (2011) Company JB (2011) Biodiversity of deep-sea demersal megafauna on western and central Mediterranean basins. Sci Mar 75: 341–350.

[16] Carney RS (2005) Zonation of deep biota on continental margins. Oceanogr Mar Biol Annu Rev 43: 211–278.

[17] Gage JD (2003) Food inputs, utilization, carbon flow and energetics. In: Tyler PA, editor. Ecosystems of the world (Ecosystems of the Deep Ocean). Amsterdam: Elsevier. 315–382.

[18] Tecchio S, Ramírez-Llodra E, Sardà F (2011) Company JB, Palomera I, et al (2011) Drivers of deep Mediterranean megabenthos communities along longitudinal and bathymetric gradients. Mar Ecol Prog Ser 439: 181–192.

[19] Carrassón M, Cartes JE (2002) Trophic relationships in a Mediterranean deep-sea fish community: partition of food resources, dietary overlap and connections within the benthic boundary layer. Mar Ecol Prog Ser 241: 41–55.

[20] Zintzen V, Anderson MJ, Roberts CD, Diebel CE (2011) Increasing variation in taxonomic distinctness reveals clusters of specialists in the deep sea. Ecography 34: 306–317.

[21] Carrassón M, Matallanas J (1998) Feeding habits of *Alepocephalus rostratus* (Pisces: Alepocephalidae) in the Western Mediterranean Sea. J Mar Biol Ass UK 78: 1295–1306.

[22] Layman CA, Araujo MS, Boucek R, Hammerschlag-Peyer CM, Harrison E, et al (2012) Applying stable isotopes to examine food-web structure: an overview of analytical tools. Biol Rev 87: 545–562. 22051097 10.1111/j.1469-185X.2011.00208.x

[23] DeNiro MJ, Epstein S (1978) Influence of diet on the distribution of carbon isotopes in animals. Geochim Cosmochim Acta 42: 495–506.

[24] DeNiro MJ, Epstein S (1981) Influence of diet on the distribution of nitrogen isotopes in animals. Geochim Cosmochim Acta 45: 341–351.

[25] McCutchan JH, Lewis WM, Kendall C, McGrath CC (2003) Variation in trophic shift for stable isotope ratios of carbon, nitrogen, and sulfur. Oikos 102: 378–390.

[26] Peterson B, Fry B (1987) Stable isotopes in ecosystem studies. Annu Rev Ecol Syst 18: 293–320.

[27] Hobson KA, Welch HE (1992) Determination of trophic relationships within a high Arctic marine food web using δ^13^C and δ^15^N analysis. Mar Ecol Prog Ser 84: 9–18.

[28] Navarro J, Coll M, Louzao M, Palomera I, Delgado A, et al (2011) Comparison of ecosystem modelling and isotopic approach as ecological tools to investigate food webs in the NW Mediterranean Sea. J Exp Mar Biol Ecol 401: 97–104.

[29] Newsome SD, Martinez del Rio C, Bearhop S, Phillips DL (2007) A niche for isotopic ecology. Front Ecol Environ 5: 429–436.

[30] Hutchinson GE (1957) Concluding remarks. Cold Spring Harbour Symposium on Quantitative Biology 22: 415–427.

[31] Bearhop S, Adams CE, Waldron S, Fuller RA, Macleod H (2004) Determining trophic niche width: a novel approach using stable isotope analysis. J Anim Ecol 73: 1007–1012.

[32] Layman CA, Arrington DA, Montaña CG, Post DM (2007) Can stable isotope ratios provide for community-wide measures of trophic structure? Ecology 88: 42–48. 17489452 10.1890/0012-9658(2007)88[42:csirpf]2.0.co;2

[33] Jackson AL, Inger R, Parnell AC, Bearhop S (2011) Comparing isotopic niche widths among and within communities: SIBER – Stable Isotope Bayesian Ellipses in R. J Anim Ecol. 80: 595–602. 10.1111/j.1365-2656.2011.01806.x 21401589

[34] Sardà F, Cartes JE (1998) Company JB, Albiol A (1998) A modified commercial trawl used to sample deep-sea megabenthos. Fish Sci 64: 492–493.

[35] Jacob U, Mintenbeck K, Brey T, Knust R, Beyer K (2005) Stable isotope food web studies: a case for standardized sample treatment. Mar Ecol Prog Ser 287: 251–253.

[36] Parnell AC, Inger R, Bearhop S, Jackson AL (2010) Source partitioning using stable isotopes: coping with too much variation. PLOS ONE 5(3): e9672. 20300637 10.1371/journal.pone.0009672 PMC2837382

[37] Costalago D, Navarro J, Álvarez-Calleja I, Palomera I (2012) Ontogenetic and seasonal changes in the feeding habits and trophic levels of two small pelagic fish species. Mar Ecol Prog Ser 460: 169–181.

[38] Post DM (2002) Using stable isotopes to estimate trophic position: models, methods, and assumptions. Ecology 83: 703–718.

[39] Sanchez-Vidal A, Canals M, Calafat A, Lastras G, Pedrosa-Pàmies R, et al (2012) Impacts on the deep-sea ecosystem by a severe coastal storm. PLOS ONE 7(1): e30395. 22295084 10.1371/journal.pone.0030395 PMC3266243

[40] Sardà F, Calafat A, Flexas M, Tselepides A, Canals M, et al (2004) An introduction to Mediterranean deep-sea biology. Sci Mar 68S3: 7–38.

[41] Smith CR, Mincks SL, DeMaster DJ (2006) A synthesis of bentho-pelagic coupling on the Antarctic shelf: food banks, ecosystem inertia and global climate change. Deep-Sea Res II 53: 875–894.

[42] Massutí E, Gordon JDM, Moranta J, Swan SC, Stefanescu C, et al (2004) Mediterranean and Atlantic deep-sea fish assemblages: differences in biomass composition and size-related structure. Sci Mar 68S3: 101–115.

[43] Ramírez-Llodra E (2010) Company JB, Sardà F, Rotllant G (2010) Megabenthic diversity patterns and community structure of the Blanes submarine canyon and adjacent slope in the Northwestern Mediterranean: a human overprint? Mar Ecol 31: 167–182.

[44] Mees J, Jones MB (1997) The hyperbenthos. Oceanogr Mar Biol Annu Rev 35: 221–255.

[45] Fanelli E, Cartes JE, Rumolo P, Sprovieri M (2009) Food-web structure and trophodynamics of mesopelagic-suprabenthic bathyal macrofauna of the Algerian Basin based on stable isotopes of carbon and nitrogen. Deep-Sea Res I 56: 1504–1520.

[46] Zuñiga D, Flexas MM, Sanchez-Vidal A, Coenjaerts J, Calafat A, et al (2009) Particle fluxes dynamics in Blanes submarine canyon (Northwestern Mediterranean). Prog Oceanogr 82: 239–251.

[47] Billett DSM, Lampitt RS, Rice AL, Mantoura RFC (1983) Seasonal sedimentation of phytoplankton to the deep-sea benthos. Nature 302: 520–522.

[48] Lampitt RS (1985) Evidence for seasonal deposition of detritus to the deep-sea floor and its subsequent resuspension. Deep-Sea Res A 32: 885–897.

[49] Smith KL, Druffel ERM (1998) Long time-series monitoring of an abyssal site in the NE Pacific: an introduction. Deep-Sea Res II 45: 573–586.

[50] Smith KL, Kaufmann RS, Baldwin RJ (1994) Coupling of near-bottom pelagic and benthic processes at abyssal depths in the eastern North Pacific Ocean. Limnol Oceanogr 39: 1101–1118.

[51] Jeffreys RM, Lavaleye MSS, Bergmann MJN, Duineveld GCA, Witbaard R (2011) Do abyssal scavengers use phytodetritus as a food resource? Video and biochemical evidence from the Atlantic and Mediterranean. Deep-Sea Res I 58: 415–428.

[52] Drazen JC, Popp BN, Choy CA, Clemente T, De Forest L, et al (2008) Bypassing the abyssal benthic food web: Macrourid diet in the eastern North Pacific inferred from stomach content and stable isotopes analyses. Limnol Oceanogr 53: 2644–2654.

[53] Sabatés A, Pagès F, Atienza D, Fuentes V, Purcell JE, et al (2010) Planktonic cnidarian distribution and feeding of *Pelagia noctiluca* in the NW Mediterranean Sea. Hydrobiologia 645: 153–165.

[54] Fry B (1988) Food web structure on Georges Bank from stable C, N, and S isotopic compositions. Limnol Oceanogr 33: 1182–1190.

[55] Nadon M-O, Himmelman JH (2006) Stable isotopes in subtidal food webs: Have enriched carbon ratios in benthic consumers been misinterpreted? Limnol Oceanogr 51: 2828–2836.

[56] Tyler PA (2003) The peripheral deep seas. In: Tyler PA, editor. Ecosystems of the world (Ecosystems of the Deep Ocean). Amsterdam: Elsevier. 261–293.

[57] Sanchez-Vidal A, Pasqual C, Kerhervé P, Heussner S, Calafat A, et al (2009) Across margin export of organic matter by cascading events traced by stable isotopes, northwestern Mediterranean Sea. Limnol Oceanogr 54: 1488–1500.

