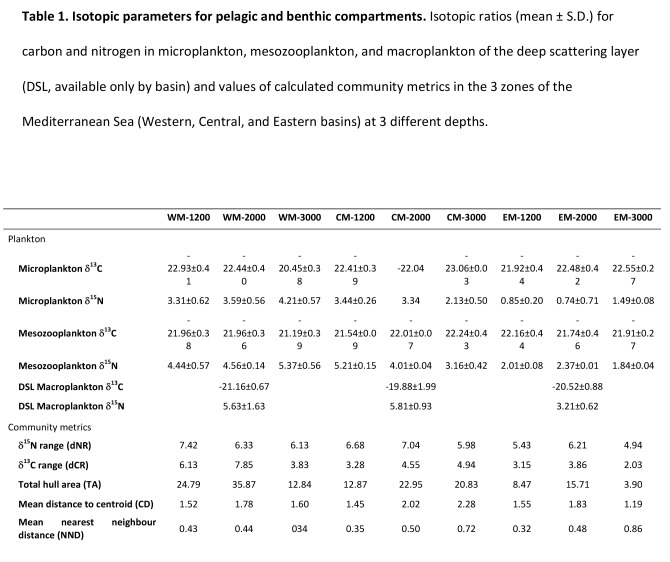# Correction: Trophic Dynamics of Deep-Sea Megabenthos Are Mediated by Surface Productivity

**DOI:** 10.1371/annotation/f4c4225c-17de-4449-9fd8-0eaa6a2dc96d

**Published:** 2014-01-17

**Authors:** Samuele Tecchio, Dick van Oevelen, Karline Soetaert, Joan Navarro, Eva Ramírez-Llodra

Table 1 was incorrectly formatted causing a series of values to be in the incorrect positions. Please see the correct Table 1 here: 

**Figure:**