# Supplementary figures and images for: Trophic Dynamics of Deep-Sea Megabenthos Are Mediated by Surface Productivity
Source: PLoS One. 2013 May 17;8(5):e63796. doi: 10.1371/journal.pone.0063796 (PMC3656946; doi:10.1371/journal.pone.0063796)

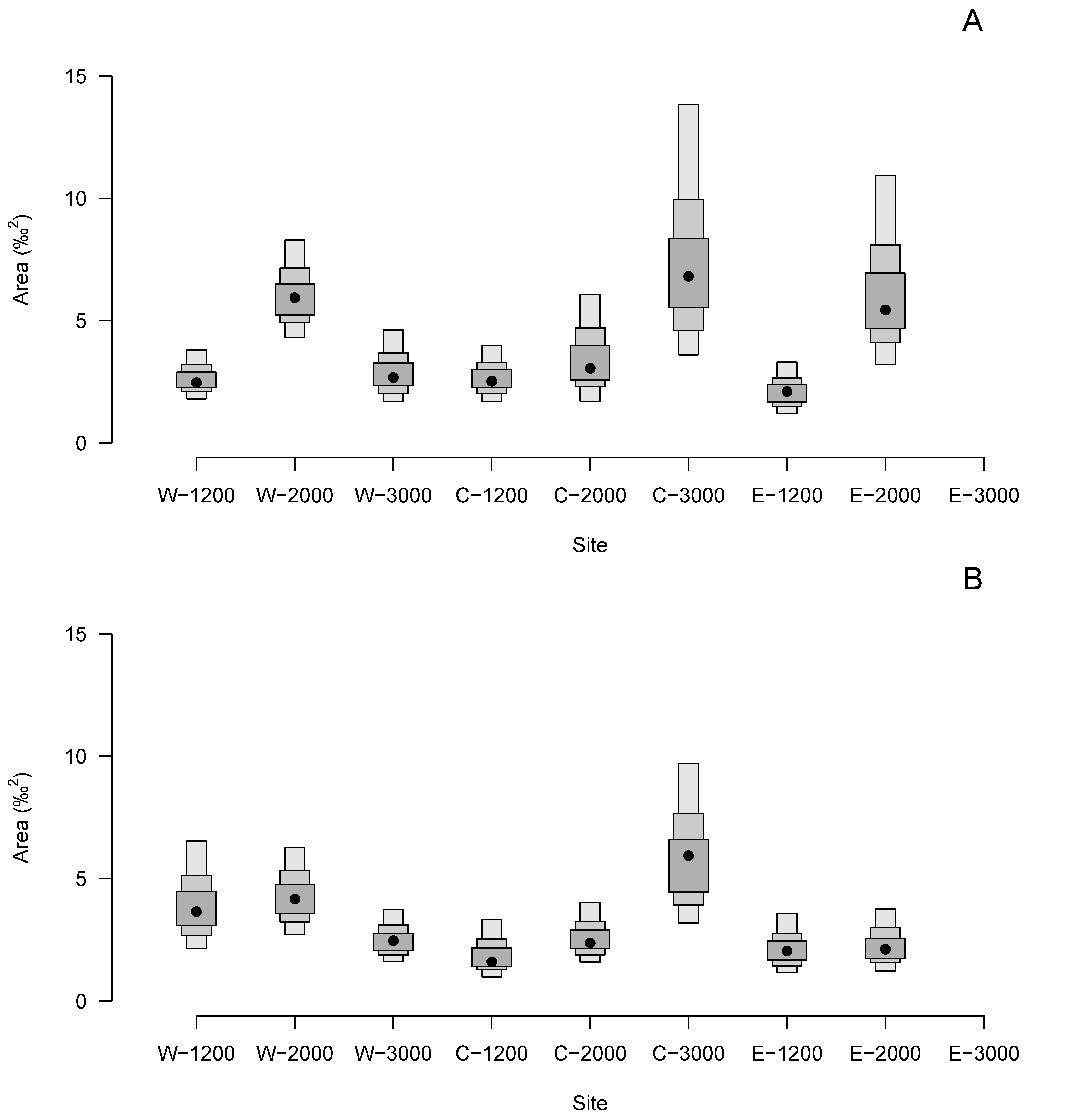

Supplement: Figure S1 — Benthic community niche widths in the deep Mediterranean Sea. Area of Bayesian isotopic ellipses for demersal fishes (A) and crustaceans (B) in the 3 basins of the Mediterranean Sea (WM, CM, EM) at 3 different depths (1200, 2000, and 3000 m). Black dots represent the simulated median. Boxes represent the 50%, 75% and 95% confidence intervals (dark grey to light grey, respectively). (TIF) [file pone.0063796.s001.tif]
