# Supplementary material for: Trophic Dynamics of Deep-Sea Megabenthos Are Mediated by Surface Productivity
Source: PLoS One. 2013 May 17;8(5):e63796. doi: 10.1371/journal.pone.0063796 (PMC3656946; doi:10.1371/journal.pone.0063796)
Supplement: Table S1 — Carbon and nitrogen isotope ratios of benthic megafauna. Values of δ13C and δ15N for all sampled benthic megafauna in the deep Mediterranean Sea (Mean ± S.D.), along with the measured carbon/nitrogen ratio (C:N) and the number of analysed specimen. (DOC) [file pone.0063796.s002.doc]

**Table S1. Carbon and nitrogen isotope ratios of benthic megafauna.** Values of δ^13^C and δ^15^N for all sampled benthic megafauna in the deep Mediterranean Sea (Mean ± S.D.), along with the measured carbon/nitrogen ratio (C:N) and the number of analysed specimen.

| **Site** | ***Species*** |  | **δ^13^C** | **δ^15^N** | **C:N** | **(n)** |
| --- | --- | --- | --- | --- | --- | --- |
| **WM-1200** |  |  |  |  |  |  |
|  | *Alepocephalus rostratus* |  | -19.12 ± 1.64 | 9.86 ± 0.19 | 4.81 | 4 |
|  | *Aristeus antennatus* |  | -17.59 ± 0.43 | 9.61 ± 0.2 | 3.67 | 4 |
|  | *Bathypterois mediterraneus* |  | -18.05 ± 0.51 | 10.97 ± 0.29 | 3.88 | 7 |
|  | *Galeus melastomus* |  | -16.93 ± 0.13 | 10.04 ± 0.21 | 3.01 | 4 |
|  | *Geryon longipes* |  | -17.29 ± 0.53 | 9.22 ± 1.16 | 3.77 | 6 |
|  | *Lepidion lepidion* |  | -17.56 ± 0.26 | 11.2 ± 0.25 | 3.89 | 4 |
|  | *Meganyctiphanes norvegica* |  | -20.53 ± 0.34 | 5.19 ± 0.57 | 4.00 | 5 |
|  | *Mora moro* |  | -17.27 ± 0.13 | 11.78 ± 0.23 | 3.80 | 4 |
|  | *Nettastoma melanurum* |  | -18.45 ± 0.49 | 11.7 ± 0.46 | 5.30 | 2 |
|  | *Nezumia sclerorhynchus* |  | -16.54 ± 0.83 | 12.22 ± 0.31 | 3.64 | 4 |
|  | *Opistoteuthis calypso* |  | -18.38 ± 0.11 | 7.67 ± 0.53 | 4.05 | 2 |
|  | *Trachyrincus scabrus* |  | -16.86 ± 0.58 | 10.25 ± 0.41 | 3.63 | 4 |
|  | *Suprabenthos* |  | -20.16 ± 0.88 | 6.39 ± 1.67 | 4.34 | 3 |
| **WM-2000** |  |  |  |  |  |  |
|  | *Acantephyra eximia* |  | -18.11 ± 0.35 | 8.35 ± 0.76 | 3.80 | 4 |
|  | *Alepocephalus rostratus* |  | -19.54 ± 1.87 | 10.59 ± 1.12 | 5.18 | 8 |
|  | *Aristeus antennatus* |  | -17.28 ± 0.4 | 10.35 ± 1.5 | 3.63 | 4 |
|  | *Bathypterois mediterraneus* |  | -17.3 ± 0.78 | 11.2 ± 0.2 | 3.83 | 4 |
|  | *Bonellia viridis* |  | -16.21 ± 2.21 | 9.18 ± 3.51 | 4.73 | 2 |
|  | *Cataetyx laticeps* |  | -18.3 ± 2.97 | 12.75 ± 0.16 | 9.66 | 5 |
|  | *Centroscymnus coelolepis* |  | -16.87 ± 0.25 | 11.35 ± 1.13 | 2.99 | 4 |
|  | *Chaceon mediterraneus* |  | -16.81 | 11.66 | 3.87 | 1 |
|  | *Chauliodus sloani* |  | -18.39 | 8.31 | 4.21 | 1 |
|  | *Coryphaenoides guentheri* |  | -16.87 ± 0.43 | 11.11 ± 0.47 | 3.76 | 5 |
|  | *Coryphaenoides mediterraneus* |  | -16.56 ± 0.41 | 10.97 ± 0.36 | 3.74 | 5 |
|  | *Lampanyctus crocodilus* |  | -20.07 | 7.59 | 4.46 | 1 |
|  | *Lepidion lepidion* |  | -16.61 ± 0.44 | 11.65 ± 0.71 | 3.63 | 5 |
|  | *Nematocarcinus exilis* |  | -17.11 ± 0.34 | 9.7 ± 0.42 | 3.83 | 4 |
|  | *Sergestes arcticus* |  | -20.71 ± 0.58 | 6.4 ± 0.75 | 3.89 | 2 |
|  | *Sergia robusta* |  | -19.85 ± 0.25 | 7.16 ± 0.48 | 3.71 | 2 |
|  | *Stereomastis sculpta* |  | -17.2 ± 0.33 | 10.94 ± 0.22 | 3.78 | 4 |
|  | *Suprabenthos* |  | -20.87 | 6.63 | 4.87 | 1 |
| **WM-3000** |  |  |  |  |  |  |
|  | *Acantephyra eximia* |  | -17.98 ± 0.47 | 8.87 ± 1.12 | 3.68 | 4 |
|  | *Acantephyra pelagica* |  | -18.28 ± 0.16 | 7.33 ± 0.34 | 3.65 | 4 |
|  | *Argyropelecus hemigymnus* |  | -20.52 | 7.80 | 4.81 | 1 |
|  | *Aristeus antennatus* |  | -17.29 | 10.53 | 3.66 | 1 |
|  | *Bathypterois mediterraneus* |  | -17.99 ± 0.33 | 11.58 ± 0.17 | 3.73 | 4 |
|  | *Centroscymnus coelolepis* |  | -17.82 ± 0.2 | 11.69 ± 0.23 | 3.31 | 2 |
|  | *Chaceon mediterraneus* |  | -17.84 ± 0.17 | 10.21 ± 0.67 | 3.81 | 3 |
|  | *Coryphaenoides guentheri* |  | -17.21 ± 0.03 | 10.74 ± 0.66 | 3.86 | 2 |
|  | *Coryphaenoides mediterraneus* |  | -17.32 ± 0.35 | 11.27 ± 0.18 | 3.66 | 4 |
|  | *Gennadas elegans* |  | -20.89 ± 1.69 | 5.48 ± 0.28 | 5.95 | 4 |
|  | *Lampanyctus crocodilus* |  | -19.77 ± 0.76 | 8.46 ± 0.44 | 4.15 | 3 |
|  | *Nematocarcinus exilis* |  | -17.1 ± 0.29 | 9.14 ± 0.24 | 3.64 | 4 |
|  | *Pelagia noctiluca* |  | -19.14 ± 0.96 | 5.32 ± 0.47 | 3.46 | 4 |
|  | *Stereomastis sculpta* |  | -17.01 ± 0.38 | 10.47 ± 0.48 | 4.29 | 4 |
|  | *Suprabenthos* |  | -19.29 | 5.72 | 4.58 | 1 |
| **CM-1200** |  |  |  |  |  |  |
|  | *Acantephyra pelagica* |  | -17.83 ± 0.19 | 6.01 ± 0.78 | 3.75 | 3 |
|  | *Argyropelecus hemigymnus* |  | -18.41 | 6.76 | 3.64 | 1 |
|  | *Aristeus antennatus* |  | -17.39 ± 0.26 | 8.34 ± 0.37 | 3.68 | 4 |
|  | *Bathypterois mediterraneus* |  | -17.9 ± 0.4 | 10.03 ± 0.62 | 3.80 | 4 |
|  | *Chauliodus sloani* |  | -17.75 ± 0.64 | 7.07 ± 0 | 3.91 | 2 |
|  | *Chimaera monstrosa* |  | -14.29 | 11.16 | 2.74 | 1 |
|  | *Coelorincus mediterraneus* |  | 15.86 | 10.28 | 3.57 | 1 |
|  | *Etmopterus spinax* |  | -17.03 ± 0.29 | 9.29 ± 1.02 | 3.00 | 3 |
|  | *Galeus melastomus* |  | -15.92 ± 1.23 | 8.14 ± 1.16 | 2.88 | 2 |
|  | *Hexanchus griseus* |  | -14.52 | 10.58 | 2.98 | 1 |
|  | *Lampanyctus crocodilus* |  | -19.58 ± 1.31 | 8.09 ± 0.47 | 5.51 | 5 |
|  | *Lepidion lepidion* |  | -16.89 ± 0.03 | 10.49 ± 0.32 | 3.78 | 2 |
|  | *Mora moro* |  | -16.59 ± 0.28 | 9.89 ± 0.26 | 3.72 | 4 |
|  | *Nettastoma melanurum* |  | -17.54 ± 1.29 | 9.8 ± 0.66 | 4.57 | 3 |
|  | *Nezumia sclerorhynchus* |  | -16.38 ± 0.46 | 11.32 ± 0.52 | 3.68 | 7 |
|  | *Notacanthus bonaparte* |  | -17.57 ± 1.42 | 8.5 ± 0.55 | 5.53 | 4 |
|  | *Phycis blennoides* |  | -16.00 | 12.51 | 3.70 | 1 |
|  | *Polycheles typhlops* |  | -17.03 ± 0.12 | 8.87 ± 0.13 | 3.62 | 4 |
|  | *Sergestes corniculum* |  | -19.81 ± 0.32 | 3.79 ± 0.44 | 3.63 | 3 |
|  | *Sergia robusta* |  | -19.13 ± 0.47 | 5.96 ± 0.68 | 3.74 | 8 |
|  | *Stomias boa* |  | -17.23 | 8.51 | 3.70 | 1 |
| **CM-2000** |  |  |  |  |  |  |
|  | *Acantephyra eximia* |  | -17.89 ± 0.19 | 6.71 ± 0.38 | 3.76 | 4 |
|  | *Acantephyra pelagica* |  | -19.25 ± 0.31 | 4.67 ± 0.9 | 4.16 | 2 |
|  | *Aristeus antennatus* |  | -17.79 ± 0.48 | 6.97 ± 0.84 | 3.81 | 6 |
|  | *Bathypterois mediterraneus* |  | -17.63 ± 0.32 | 10.23 ± 0.47 | 3.69 | 6 |
|  | *Coryphaenoides guentheri* |  | -15.97 | 10.62 | 3.73 | 1 |
|  | *Coryphaenoides mediterraneus* |  | -18.69 ± 1.61 | 18.6 ± 1.8 | 3.72 | 2 |
|  | Isopoda sp. |  | -18.46 | 6.46 | 4.43 | 1 |
|  | *Lampanyctus crocodilus* |  | -20.46 | 5.57 | 4.37 | 1 |
|  | Leptocephalus larvae |  | -21.6 ± 0.16 | 3.91 ± 0.93 | 5.04 | 2 |
|  | *Lepidion lepidion* |  | -18.34 | 6.97 | 3.62 | 1 |
|  | *Nematocarcinus exilis* |  | -17.62 ± 0.44 | 5.66 ± 0.28 | 4.01 | 4 |
|  | *Suprabenthos* |  | -20.52 | 4.61 | 5.32 | 1 |
| **CM-3000** |  |  |  |  |  |  |
|  | *Acantephyra eximia* |  | -18.01 ± 0.18 | 7.38 ± 0.84 | 3.86 | 4 |
|  | *Bathypterois mediterraneus* |  | -18.15 | 10.87 | 3.95 | 1 |
|  | *Cataetyx laticeps* |  | -18.79 ± 1.28 | 10.85 ± 0.3 | 6.10 | 3 |
|  | *Chaceon mediterraneus* |  | -18.77 ± 1.74 | 10.15 ± 0.87 | 5.76 | 4 |
|  | *Chauliodus sloani* |  | -19.10 | 5.03 | 3.95 | 1 |
|  | *Coryphaenoides guentheri* |  | -15.98 ± 0.09 | 11.04 ± 0.46 | 3.78 | 2 |
|  | *Coryphaenoides mediterraneus* |  | -18.2 ± 1.68 | 8.5 ± 2.79 | 3.79 | 3 |
|  | *Melanostigma atlanticum* |  | -18.56 | 8.51 | 4.40 | 1 |
|  | *Nematocarcinus exilis* |  | -17.98 ± 0.55 | 6.16 ± 0.56 | 3.77 | 4 |
|  | *Phronima sedentaria* |  | -19.13 | 3.27 | 4.47 | 1 |
|  | *Pyrosoma atlanticum* |  | -21.75 ± 0.05 | 1.59 ± 0.06 | 6.14 | 3 |
| **EM-1200** |  |  |  |  |  |  |
|  | *Acantephyra eximia* |  | -17.78 ± 0.31 | 4.89 ± 0.26 | 3.75 | 2 |
|  | *Argyropelecus hemigymnus* |  | -19.67 | 3.66 | 3.80 | 1 |
|  | *Aristeus antennatus* |  | -17.45 ± 0.16 | 6.28 ± 0.07 | 3.89 | 4 |
|  | *Bathypterois mediterraneus* |  | -17.48 ± 0.64 | 7.68 ± 0.3 | 3.68 | 6 |
|  | *Dalatias licha* |  | -16.79 | 7.81 | 2.97 | 1 |
|  | *Galeus melastomus* |  | -16.35 | 7.72 | 2.77 | 1 |
|  | *Mora moro* |  | -16.34 | 8.81 | 3.79 | 1 |
|  | *Nettastoma melanurum* |  | -16.71 | 7.72 | 4.15 | 1 |
|  | *Nezumia sclerorhynchus* |  | -16.93 ± 0.42 | 9.41 ± 0.9 | 3.80 | 7 |
|  | *Plesionika acantonotus* |  | -17.24 ± 0.2 | 5.06 ± 0.44 | 3.66 | 3 |
|  | *Polycheles typhlops* |  | -17.13 ± 0 | 7.3 ± 0.09 | 3.72 | 2 |
|  | *Pyrosoma atlanticum* |  | -21.39 ± 0.16 | 1.22 ± 0.7 | 7.26 | 2 |
|  | *Sergia robusta* |  | -19.28 | 3.39 | 3.62 | 1 |
|  | *Suprabenthos* |  | -19.41 | 5.01 | 4.39 | 1 |
| **EM-2000** |  |  |  |  |  |  |
|  | *Acantephyra eximia* |  | -17.26 ± 0.74 | 5.84 ± 0.35 | 3.70 | 4 |
|  | *Argyropelecus hemigymnus* |  | -18.62 | 4.89 | 3.63 | 1 |
|  | *Aristeus antennatus* |  | -17.4 ± 0.11 | 7.06 ± 0.47 | 3.82 | 2 |
|  | *Bathypterois mediterraneus* |  | -17.85 ± 0.63 | 7.84 ± 2.1 | 3.76 | 5 |
|  | *Cataetyx laticeps* |  | -20.03 ± 0.29 | 8.86 ± 0.34 | 10.14 | 3 |
|  | *Coryphaenoides mediterraneus* |  | -16.50 | 7.27 | 3.66 | 1 |
|  | Leptocephalus larvae |  | -21.04 | 1.40 | 5.39 | 1 |
|  | *Lepidion lepidion* |  | -18.44 ± 1.06 | 6.92 ± 2.25 | 3.87 | 2 |
|  | *Nematocarcinus exilis* |  | -17.91 ± 0.48 | 4.36 ± 0.29 | 4.24 | 5 |
|  | *Pasiphaea multidentata* |  | -17.76 | 4.52 | 3.72 | 1 |
|  | *Polycheles typhlops* |  | -16.8 | 6.17 | 3.57 | 1 |
|  | *Sergestes corniculum* |  | -19.24 ± 0.31 | 2.43 ± 0.1 | 3.83 | 3 |
| **EM-3000** |  |  |  |  |  |  |
|  | *Acantephyra eximia* |  | -18.38 ± 0.31 | 5.58 ± 0.39 | 3.92 | 4 |
|  | *Coryphaenoides mediterraneus* |  | -16.6 | 8.36 | 3.79 | 1 |
|  | *Nematocarcinus exilis* |  | -17.88 ± 0.41 | 4.18 ± 0.61 | 4.06 | 4 |
